# The multifaceted inducers of cellular senescence

**DOI:** 10.18632/aging.206391

**Published:** 2026-06-22

**Authors:** Hilah Gal, Valery Krizhanovsky

**Affiliations:** 1 Department of Molecular Cell Biology, Weizmann Institute of Science, Rehovot 7610001, Israel

**Keywords:** aging, cell senescence

## Abstract

Cellular senescence is a stable form of cell-cycle arrest induced by diverse intrinsic and extrinsic stimuli. While senescence contributes to tumor suppression, wound repair, and placental and embryonic development, the chronic accumulation of senescent cells promotes tissue dysfunction, chronic inflammation, tumorigenesis, and age-related diseases. This review provides a comprehensive overview of the major inducers of cellular senescence, including DNA damage, oxidative and mitochondrial stress, telomere attrition, oncogene activation, cell–cell fusion, senescence-induced senescence and developmental stimuli, and summarizes the molecular mechanisms through which they trigger the senescence program. Although these stimuli differ widely, many converge to core effector pathways, resulting in a stable growth arrest. Understanding the varied stimuli and their underlying mechanisms of senescence induction is crucial for revealing the heterogeneity of senescent cells and developing interventions that modulate senescence during aging and disease.

## INTRODUCTION

Cellular senescence is a fundamental biological program in which cells undergo stable and long-term cell-cycle arrest while remaining metabolically active [1–3]. Induction of cellular senescence functions as a potent tumor-suppressive mechanism, preventing the proliferation of damaged or potentially cancer-prone cells. It also fulfills essential physiological functions during embryonic development, placental maturation, wound repair, and tissue remodeling, illustrating its roles in normal organismal biology [4–11]. In young individuals, senescent cells are efficiently cleared by the immune system, thus maintaining their homeostatic turnover. With advancing age, however, declining immune surveillance and impaired turnover lead to the progressive accumulation of senescent cells in tissues [12]. The chronic presence of senescent cells is detrimental, largely due to the senescence-associated secretory phenotype (SASP), which promotes inflammation, disrupts tissue homeostasis, and contributes to tumorigenesis, tissue dysfunction, and multiple age-related diseases [13–16].

At the molecular level, senescence is enforced and maintained primarily through the p53/p21 and p16/Rb tumor-suppressor pathways, which coordinately establish cell-cycle arrest [2, 3]. These pathways, together with persistent DNA damage response (DDR), also influence the development and composition of the SASP, a complex secretome of cytokines, chemokines, growth factors, and proteases that mediates many of the non-autonomous effects of senescent cells [13–16]. Regulatory mechanisms such as DNA-damage signaling, chromatin remodeling, metabolic rewiring, and persistent stress responses modulate both the induction of senescence and the qualitative features of the SASP, linking the core senescence machinery to downstream tissue-level consequences.

Senescence is a highly heterogeneous phenotype, and this heterogeneity arises from several layers of biological diversity [17]. Different cell types may vary in their susceptibility to enter senescence and in the molecular pathways they activate upon entering this state, in addition to the core cell-cycle arrest machinery [17–19]. This context-dependent variability is pronounced, such that senescent cells do not share a universal molecular signature, necessitating the use of multiple markers for their accurate identification [20, 21]. Microenvironmental conditions, including inflammatory cues, extracellular matrix composition [22], oxygen levels, and immune context, further shape the senescence response and SASP [13–16]. Moreover, distinct senescence-inducing stimuli may engage overlapping but not identical signaling networks, leading to variation in gene-expression profiles, metabolic changes [23], and secretory programs [2, 3]. Together, these factors can create a spectrum of senescent cell phenotypes that differ in their impact on tissue physiology [8, 24].

In this review, we focus on the major inducers of cellular senescence. While well-established inducers such as DNA damage and oxidative stress are central drivers of senescence in aging and disease, we also discuss physiological and context-specific triggers to provide a more comprehensive and integrative perspective on senescence induction. Starting from the first-described form of senescence by Hayflick, replicative senescence associated with prolonged cell culture [25], we survey the major triggers that activate the senescence program through diverse molecular mechanisms (Figure 1). Integrating insights into these distinct stimuli, the signaling pathways they engage, and their functional consequences might help to clarify how distinct populations of senescent cells contribute to aging, cancer, and age-related pathologies, and assist in the development of new therapeutic strategies aimed at modulating senescence and its deleterious consequences without deteriorating its beneficial functions.

**Figure 1:**
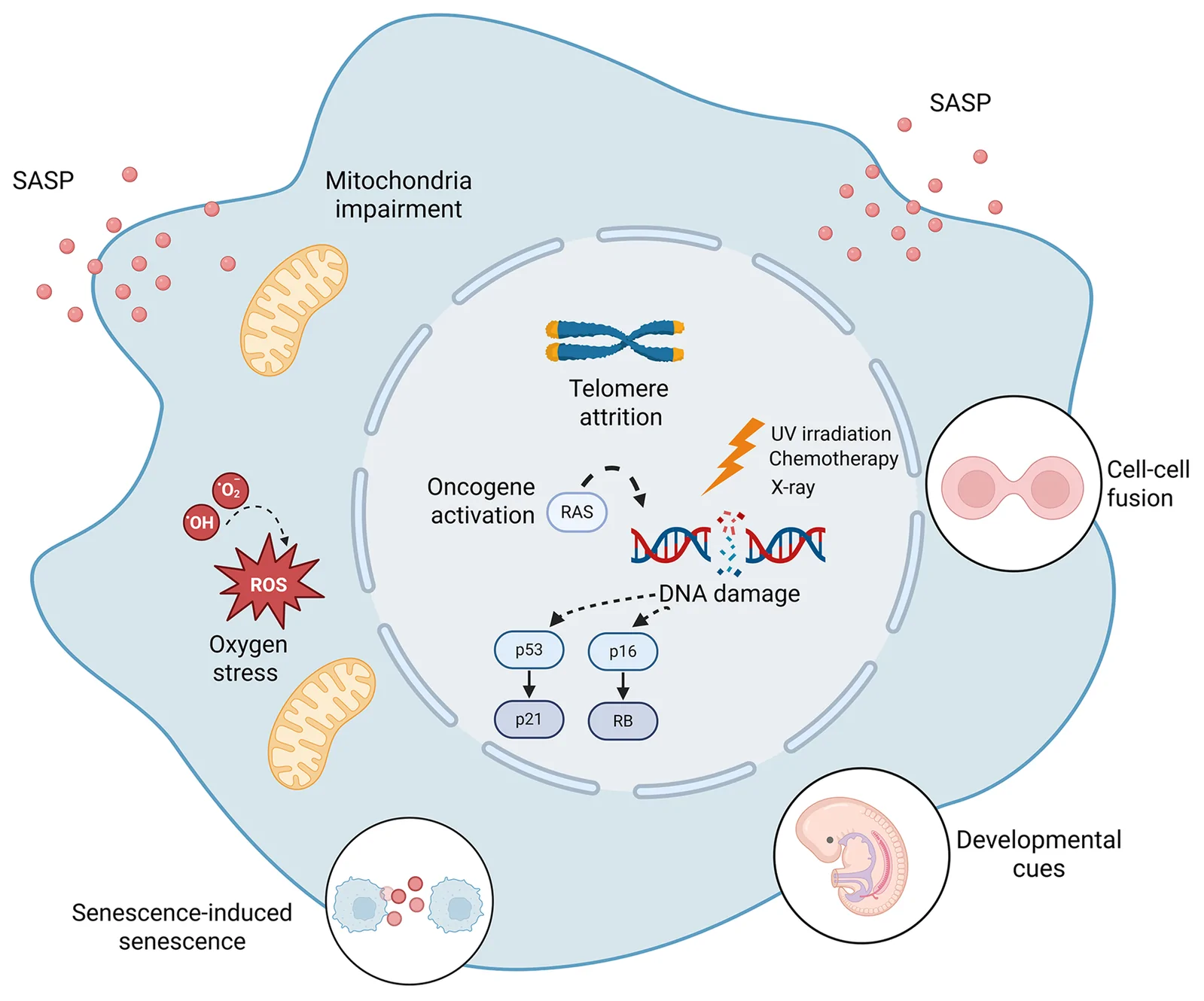
**Major inducers of cellular senescence.** Diverse intrinsic and extrinsic stimuli, including DNA damage, oncogene activation, telomere attrition, oxidative stress, mitochondrial impairment, cell-cell fusion, inflammatory signals, and developmental cues, initiate cellular senescence through distinct mechanisms. These stimuli converge on shared effector pathways, including persistent DNA damage response (DDR) signaling and activation of the p53/p21 and p16/Rb pathways, resulting in stable growth arrest and context-dependent SASP programs.

## Replicative senescence and telomere attrition

Replicative senescence was first reported by Hayflick and Moorhead in 1961, who observed that normal human fibroblasts reach a finite number of cell divisions [25]. The molecular basis of this phenomenon, also termed the “Hayflick limit”, is primarily due to the progressive shortening of telomeres, resulting in gradual attrition, ultimately triggering a DDR, followed by senescence induction. Telomeres are tandem repeats of nucleotide sequences (TTAGGG in vertebrates) located at the terminals of linear chromosomes. Binding of specialized protein complexes known as “Shelterin”, comprised of six proteins: telomeric repeat binding factor 1 (TRF1), telomeric repeat binding factor 2 (TRF2), TRF2 interacting protein (RAP1), TRF1-interacting nuclear factor 2 (TIN2), adrenocortical dysplasia protein homolog (TPP1) and protection of telomeres 1 (POT1), protect chromosomal ends from being recognized as DNA double-strand breaks [26]. This complex binds telomeric DNA and facilitates its folding into a protective three-dimensional T-loop structure that safeguards the 3′–OH terminus [27]. Additionally, it prevents unwanted DNA repair activities such as end-to-end fusions or chromosomal degradation, thus maintaining genomic stability [26, 28].

During DNA replication, there is a progressive loss of telomere repeats, also referred to as the “*end-replication problem”*, leading to gradual telomere shortening [29], which correlates with senescence induction [30]. Once telomeres reach a critically short length, they fail to sufficiently bind Shelterin components, resulting in the exposure of telomere ends, recognized by the DNA repair machinery as double-strand DNA breaks, thus triggering DNA damage responses and contributing to cellular senescence.

Deletion of Shelterin component TRF2 in human cells results in the activation and recruitment of proteins involved in DDR, including 53BP1, the Mre11 complex and phosphorylated forms of ATM, H2A.X and Rad 17 [31]. Similarly, conditional deletion of the Pot1a Shelterin component in mice results in the activation of a DDR, specifically at telomere regions and induction of senescence [32]. Moreover, replicative senescent human fibroblasts accumulate proteins involved in the DDR at telomere regions, including γH2A.X, 53BP1, MDC1 and NBS1 [33]. Interestingly, mild oxidative stress can also induce breaks within telomeric regions, accelerating telomere shortening and senescence. Telomere dysfunction can also occur independently of shortening, whereby damage arising within telomeric repeats triggers DDR signaling and senescence [34].

Telomerase, a ribonucleoprotein enzyme complex, counteracts telomere shortening driven by cell division and bypasses the senescence arrest [35] by appending the repetitive sequences to chromosome ends [36]. It consists of a catalytic subunit, telomerase reverse transcriptase (TERT), and an RNA template (TERC) that directs telomeric DNA synthesis [37]. While most somatic cells exhibit low telomerase activity, certain cell types—such as germ cells, stem cells, and cancer cells—maintain high telomerase activity to support continuous proliferation [38]. Altogether, it is suggested that telomere-associated DNA damage responses play a causal role in senescence induction.

## DNA damage induced senescence

DNA damage, triggered by ionizing radiation (IR), UV light, chemotherapeutic drugs and pathological increases in intracellular and extracellular reactive oxygen species (ROS) are potent inducers of the senescence program. Senescence induced by DNA damage is entirely dependent upon a DDR [39]. Single-stranded DNA and/or the generation of DNA double-strand breaks (DSBs) are powerful activators of the DDR, via the recruitment and activation of two large protein kinases, ataxia telangiectasia and Rad3-related (ATR) or ataxia-telangiectasia mutated (ATM), respectively, at the site of DNA lesions [39, 40]. ATM/ATR targets protein kinases CHK1 and CHK2 which, together with ATM and ATR, and other adaptor proteins (p53BP1 and MDC1) and chromatin modifiers (such as γ-H2AX), act to reduce cyclin-dependent kinase (CDK) activity by various mechanisms [41]. Many of these proteins are localized to DNA-damage foci that can be detected in senescent cells [42, 43].

During senescence, DDR frequently remains chronically active, particularly at sites harboring persistent lesions such as irreparable double-strand breaks (DSBs) or dysfunctional telomeric ends [44]. These stable DDR foci play a central role in maintaining senescence-associated growth arrest and reinforcing the SASP [3]. Their persistence leads to activation of the ATM and ATR followed by p53–p21 and p16–Rb pathways, which together establish and sustain stable cell-cycle arrest characteristic of senescent cells and SASP.

There are several triggers that cause direct DNA damage and thus activate DDR. Ionizing radiation (IR), one such trigger of the DDR, induces a spectrum of DNA lesions including base modifications, single-strand breaks (SSBs), and, most notably, DSBs. In parallel, IR generates ROS, which further amplifies genomic instability by promoting oxidative DNA damage [40]. Many cytotoxic anticancer therapies induce a form of cellular senescence known as therapy-induced senescence (TIS). These agents cause substantial DNA damage not only on tumor cells but also on surrounding normal cells [45–48]. Chemotherapeutic drugs such as doxorubicin, etoposide, cisplatin, and various antimetabolites generate DNA breaks, crosslinks, replication stress, and oxidative lesions, collectively activating the DDR and enforcing a stable senescence-associated growth arrest [49].

TIS represents a major clinically relevant form of senescence and is characterized by durable proliferative arrest and a constitutive, dynamic SASP expression [50]. Although TIS arises in only a subset of treated cells, accumulating evidence indicates that these cells can profoundly influence the tumor microenvironment through paracrine signaling. In this context, TIS can exert a dual effect: while initially acting as a tumor-suppressive mechanism by halting proliferation of damaged cells, the long-term persistence of senescent cells and their SASP can promote tumor cell proliferation, migration, invasion, angiogenesis, immune modulation, and metastatic dissemination [49, 50]. Moreover, TIS cells have been implicated in therapy resistance and tumor relapse, largely through SASP-mediated effects and cellular plasticity [49, 50].

Notably, the senescent phenotypes arising in response to different chemotherapeutic agents are highly heterogeneous, indicating that although DDR activation is a shared initiating mechanism, the resulting senescence state is shaped by treatment context, drug dose, and intrinsic cell-type–specific responses [50, 51].

These observations highlight TIS as both a beneficial outcome of anticancer therapy and a potential driver of disease progression, underscoring growing interest in therapeutic strategies aimed at selectively eliminating TIS cells or modulating their environment. Understanding the molecular and environmental determinants that govern these opposing outcomes is therefore essential for optimizing the clinical benefit of TIS.

## Oxidative stress, ROS and mitochondrial dysfunction

Oxidative stress, primarily mediated by excessive production of ROS, is a potent and well-characterized inducer of cellular senescence, acting through interconnected pathways involving DNA damage, telomere dysfunction, mitochondrial impairment, and SASP activation [52, 53]. ROS are natural metabolic by-products and serve essential roles in intracellular signaling. However, their accumulation contributes to both the induction and long-term stabilization of senescence [2, 53, 54].

ROS are particularly effective at destabilizing guanine-rich telomeric repeats, accelerating telomere shortening and inducing premature senescence [55]. Interestingly, ROS can damage DNA directly, via oxidative base modifications, single-strand breaks, and double-strand breaks, thereby activating the DDR and inducing senescence [56–58]. Interestingly, persistent DDR signaling at telomeres, termed telomere dysfunction–induced foci (TIFs), has been repeatedly observed in ROS induced senescence [44, 52].

Beyond their genotoxic effects, ROS activate p38 MAPK and JNK, which enhance the stability and transcriptional activity of senescence regulators p53, p21, and p16, thereby reinforcing cell-cycle arrest [53]. ROS also potentiate NF-κB signaling, stimulating SASP secretion and further amplifying oxidative stress in an autocrine and paracrine manner [15, 53].

Senescent cells frequently exhibit increased mitochondrial mass, altered membrane potential, and disrupted cristae, which elevate ROS production and contribute to mitochondrial dysfunction [59]. Mitochondrial dysfunction-associated senescence (MiDAS) occurs when perturbations such as loss of sirtuins SIRT3, SIRT5, depletion of mitochondrial DNA, inhibition of the electron transport chain, or loss of mitochondrial chaperones induce senescence. MiDAS features a distinct SASP lacking IL1R-dependent proinflammatory cytokines, highlighting the plasticity of SASP [59, 60]. Mechanistically, mitochondrial impairment lowers the NAD+/NADH ratio and activates AMPK, leading to p53-mediated growth arrest while suppressing NF-κB-dependent SASP signaling [60, 61]. Pyruvate supplementation restores NAD+/NADH balance, aspartate synthesis, and IL1R-dependent SASP and enables proliferation of human primary fibroblasts [59, 60, 62]. Increased mitochondrial ROS further enhance DNA damage and DDR signaling, reinforcing senescence and SASP activation [58]. Notably, senescent cells accumulate in murine models of mitochondrial dysfunction and premature aging, supporting mitochondrial dysfunction as a driver of senescence *in vivo* [60, 63, 64].

Senescence has been increasingly linked to ferroptosis resistance, iron accumulation and lysosomal dysfunction, suggesting a new means of regulation in senescent-cell survival [65–67]. In particular, senescent cells can accumulate iron within lysosomes, a process associated with the expression of SASP [65] and display altered lysosomal homeostasis, which may reduce lipid peroxidation and protect these cells from ferroptotic death [66]. This link between senescence and ferroptosis implies that lysosomal alterations are not just a consequence of senescence but may actively shape senescent-cell persistence and be utilized for their elimination [67, 68]. Additionally, retrograde mitochondrial-to-nuclear signaling pathways distinct from MiDAS have been identified, including neuronal mitochondrial dysfunction that drives glial senescence through ROS and mtDNA release [69]. These mechanisms reveal how mitochondrial stress propagates senescence non-cell-autonomously via nuclear reprogramming, distinct from classical MiDAS [69].

## Oncogene induced senescence (OIS)

Oncogene-induced senescence (OIS) refers to a durable cell-cycle arrest triggered by aberrant oncogenic signaling independently of telomere length. It functions as a potent intrinsic tumor-suppressive barrier, halting proliferation in cells experiencing oncogene-activating genomic alterations [70–76]. The phenomenon was first described when expression of the constitutively active *HRAS^G12V^* oncogene in primary human fibroblasts induced the classic senescence morphology along with robust upregulation of p53 (Trp53), p21 (Cdkn1a), and p16 (Cdkn2a), ultimately enforcing irreversible growth arrest [72]. Later studies demonstrated that OIS is not restricted to oncogenic RAS. A variety of hyperactive signaling modules—including those within the RAS/MAPK pathway (e.g. *H-RAS^G12V^*, *N-RAS^Q61R^* and *B-RAF^V600E^*) and PI3K/AKT pathway (e.g. *PIK3CA^H1047R^*, *AKT1^E17K^* and *PTEN* loss), can trigger OIS across diverse cellular settings [70].

*In vivo* studies subsequently confirmed the biological relevance of OIS. In a transgenic murine *Kras^G12V^*-driven lung cancer model, senescence markers were readily detected in premalignant adenomas but lost upon progression to malignant adenocarcinoma [73]. Likewise, benign human melanocytic nevi harboring *BRAF^V600E^* exhibit a stable cell-cycle arrest [77], although these lesions often display mosaic p16 expression and limited p53 activation, suggesting that alternative senescence-inducing pathways are engaged. Further evidence emerged from lymphoma models in which loss of the chromatin modifier enzyme Suv39h1, required for Rb-mediated transcriptional repression, abrogated the OIS response and enabled aggressive tumor development upon Ras activation [78]. Similarly, prostate-specific deletion of murine *Pten* resulted in benign prostatic intraepithelial neoplasia (PIN) with a significant increase in SA-βGal staining positive cells (compared to wild-type mice). Subsequent loss of *Trp53* in *Pten*-null mice led to loss of senescence markers and invasive prostate cancer [79].

Interestingly, OIS might be mediated, at least partially, by the induction of DDR [75, 76, 80]. In fact, the expression of the activated oncogene *H*-*RAS ^G12V^* in normal human cells was shown to be a consequence of the activation of robust DDR [75]. Elevated ROS levels contribute to oxidative DNA damage, thereby reinforcing DDR activation. However, the contribution of DDR to OIS *in vivo* is not completely understood and requires further characterization. Moreover, mutant oncogenes, for example *H*-*ras ^G12V^*, have the potential to activate molecular pathways of cell senescence such as p38 and NF-kB independent of DNA damage. In addition, oncogenic Ras can promote the up-regulation of p53 via p19ARF independent of DNA damage in mice [81]. Therefore, the induction of cell senescence in the absence of DNA damage cannot be excluded.

Collectively, OIS represents a complex tumor-suppressive program initiated by excessive mitogenic signaling. Oncogenes elicit a network of intracellular signals, including replication stress, DDR activation, ROS accumulation, and activation of signaling pathways such as p38, NF-κB, and p19ARF which lead to the p53/p21 and p16/Rb activation, imposing cell-cycle arrest and activating SASP. Understanding the modular architecture of OIS, and why certain premalignant lesions sustain senescence while others escape it, remains crucial for leveraging senescence as a therapeutic strategy and for interpreting senescence biomarkers in human tumors.

Cellular senescence is accompanied by broad chromatin remodeling, including altered chromatin accessibility, repressive and active histone-mark distributions, and changes in nuclear architecture [82, 83]. While senescence-associated chromatin changes in senescent cells were initially described as features of OIS [84, 85], recent findings support the idea that senescence-associated heterochromatin remodeling, including SAHF-like features and lamin-linked nuclear changes, represents a common downstream event of diverse senescence inducers, rather than a feature limited to a specific trigger [82].

## Senescence induced senescence (SIS)

Senescence has long been considered a cell-autonomous response to damage or various cell stress stimuli. However, accumulating evidence demonstrates that senescent cells can propagate senescence to neighboring or distant cells through paracrine signaling and direct cell–cell interactions. This process, termed senescence-induced senescence (SIS), might contribute to tissue dysfunction, chronic inflammation, organismal aging and disease [86, 87]. SIS is largely driven by the senescence-associated secretory phenotype (SASP). Central SASP components, including IL-6, IL-8, TGF-β, GM-CSF, and MMPs, can induce DDR and cell-cycle arrest in neighboring cells [15]. Through these factors, SASP secretion amplifies inflammation, further inducing senescence in a self-reinforcing cycle [15].

SIS was formerly described in DNA-damaged human fibroblasts *in vitro*, where senescent cells triggered DDR activation in neighboring proliferating cells via pro-inflammatory and pro-oxidant signals, notably ROS [88]. ROS-dependent SIS has been demonstrated in both fibroblast and epithelial models [15, 54, 88]. Mitochondrial dysfunction in senescent cells enhances ROS production and activates NF-κB, which drives the expression of SASP components, including pro-inflammatory cytokines and ROS-inducing factors, thereby increasing oxidative and replicative stress and promoting DNA damage and senescence in neighboring cells [15, 54]. SIS can also be mediated by direct autocrine, juxtacrine signaling. During OIS, activation of NOTCH1 drives a TGF-β-rich secretome and promotes SIS through a contact-dependent NOTCH–JAG1 signaling pathway [89].

Inflammation-induced senescence (IIS) is a phenomenon in which chronic exposure to pro-inflammatory cytokines and chemokines drive cells into senescence, even in the absence of primary DNA damage [61, 90]. Prolonged inflammatory signaling elevates ROS, impairs mitochondrial function, and activates NF-κB and p38 MAPK pathways, ultimately inducing senescence [91]. IIS therefore acts as both a driver and amplifier of SIS. Chronic inflammation elevates senescence burden, increasing the number of SASP-secreting cells [19], while SASP-mediated inflammation in turn extends senescence propagation in a self-reinforcing cycle [15]. Together, SIS and IIS reflect a shared principle whereby elevated inflammatory signaling accelerates senescence induction and promotes senescence propagation across tissues.

## Fusion induced senescence (FIS) and placental development

Cell–cell fusion is a fundamental biological process that contributes to normal development, such as myoblast fusion during muscle formation and trophoblast fusion in the placenta. However, fusion events also occur pathologically during viral infection, inflammation, or tumor progression, resulting in multinucleated cells also known as syncytia.

Cell-cell fusion has been identified as an inducer of cellular senescence [4]. Cell Fusion-induced senescent cells (FIS) exist within a normally developed placenta, within the syncytiotrophoblast, an area where physiological cell-cell fusion occurs, thereby suggesting that cellular senescence plays a role in placental and embryonic development. Moreover, endogenous and exogenous fusogenic proteins might lead to abnormal, illicit, cell fusion. Normal and cancer cells undergoing cell-cell fusion triggered by such fusogens, such as the endogenous retroviral placental protein ERVWE1 or the measles virus, display characteristics of cellular senescence, including flat enlarged morphology and the expression of SA-β-gal [4]. In addition to cell-cycle arrest following FIS, the main molecular pathways of cellular senescence, the p53-p21 and p16-pRb pathways are activated in these settings. Other components of the senescence phenotype, such as elevation in ROS and DNA damage, are also present in these cells. FIS cells express factors of the SASP including IL-6, IL-8, CCL5 and CXCL1 that promote the interaction with the immune system. Additionally, FIS cells up-regulate the expression of ligands of activating NK cell receptor NKG2D, supporting immune surveillance [4]. Similar mechanisms are employed by the immune system to control the presence of senescent cells in cancer and in tissue damage restriction, implying that FIS has evolved as a protective mechanism to prevent the spread of infection and cancer and eliminate these threats by immunosurveillance [4, 92].

During development, FIS is also induced in the placenta by the endogenously expressed ERVWE1 fusogen [4, 5]. ERVWE1, a fusogen of viral origin, mediates cell-fusion-induced senescence of the syncytiotrophoblast, following cytotrophoblast differentiation. This multinucleated structure serves as a maternal–fetal interface, supporting fetal growth and development. Senescence in the placenta shares features of senescence induced by DNA damage and exhibits a coordinated activation of p53/p21 and p16/pRb regulatory pathways [4, 5]. Furthermore, knockout mice deficient in the main regulatory pathways of senescence exhibit functional and morphological aberrations in placental syncytiotrophoblasts [5]. Interestingly, human placentas from pregnancies with intrauterine growth restriction (IUGR) pathology exhibit reduced activation of senescence related pathways [5].

Altogether, it is suggested that FIS plays a dual role at different stages of life. During embryonic development, FIS plays a physiological role in placental development and functionality, while in the adult organism, the same mechanism is employed to protect the organism from the detrimental effects of abnormal expression of fusogens and viruses, preventing the spread of infection and cancer and facilitating their elimination by the immune system.

## Developmental senescence: a programmed role in embryogenesis

Senescence has been initially viewed as a cellular stress response mechanism. However, from an evolutionary point of view, cellular mechanisms beneficial to the organism have to be selected. Several reports have shown that senescence can play a more physiological role, with the first indications of its physiological context in wound healing responses [9, 93]. Eventually, the essential physiological role of senescence during embryonic development was discovered [4, 6, 7].

Developmental senescence is defined as a transient, programmed form of cellular senescence that occurs during embryogenesis and plays an essential and constructive role in tissue remodeling, organ patterning and morphogenesis [6, 7]. It occurs throughout embryonic development, in particular murine organs, including the endolymphatic sac of the inner ear, the regressing mesonephric tubules, the Apical Ectodermal Ridge (AER) during limb formation, the neural roof plate and other transient, embryonic tissues [6, 7].

Unlike the damage-induced senescence program, which often involves the p16/Rb and p53/p21 pathways in response to stress stimuli, developmental senescence primarily relies on the expression of p21, acting downstream of TGF-B/ SMAD and PI3K-FOXO signaling pathways, and is p53 and DDR independent. The independence of DDR underscores its constructive nature in embryogenesis. Embryonic senescent cells are transient, recruited for tissue-remodeling, and are efficiently and timely removed by the immune system, particularly by the macrophage subpopulation [6, 7].

The inducers involved in programmed developmental senescence constitute precise molecular cues that dictate cell fate and orchestrate tissue patterning and embryogenesis in a timely, regulated manner. These triggers differ in part from the well-established inducers of telomere attrition, DNA damage, oncogene activation of senescence in aging, cancer and age-related pathologies, highlighting its functional distinction. In the mesonephros and the endolymphatic sac of the inner ear, the induction was shown to be dependent on developmental signaling pathways (TGF-B/ SMAD and PI3K-FOXO) and perhaps some developmental signaling cues which are currently undetermined. Taken together, developmental senescence exhibits its own characteristic signature, while sharing certain—but not all—regulatory pathways of senescence in the adult organism.

## Recent advances and concluding remarks

In the last decade, single cell analysis technologies have reshaped our understanding of cell identity, including the view on senescent cells. Single-cell studies have shown that SASP composition is highly heterogeneous across senescence inducers, cell types, and tissues, challenging the idea of a uniform secretory program [17, 94, 95]. Bulk transcriptomic analyses further indicate that senescence gene expression programs vary by inducer and cell type [96]. scRNA-seq–based approaches have been developed to identify senescent cells and infer SASP signatures from transcriptomic data, yet these methods may not fully reflect the functional protein-level secretome, mostly due to post-transcriptional and post-translational regulation [17, 94, 95]. These analyses show that senescent populations can contain distinct substates with different inflammatory signatures, marker profiles, and responses to intervention, even within the same tissues. This emerging single-cell view emphasizes that senescence should be considered as a context-dependent state rather than a single phenotype across tissues, with important implications for biomarker selection and therapeutic targeting.

Several important controversies remain in senescence research. First, while DDR is central to many senescence programs, its necessity for OIS *in vivo* remains debated, as some models suggest DDR-independent pathways, including p38MAPK, may dominate in certain contexts [21, 97]. Second, the distinctiveness of MiDAS versus oxidative stress-induced senescence remains unclear; whether MiDAS represents a unique program or merely a metabolic variant requires additional *in vivo* validation [98]. Indeed, mitochondrial dysfunction is increasingly recognized as a core mechanism linking senescence, ferroptosis susceptibility, lysosomal remodeling, and retrograde mitochondrial-to-nuclear signaling pathways that extend beyond MiDAS, highlighting the need to distinguish MiDAS from other oxidative stress- and metabolism-driven senescence programs [98]. Third, although SIS and IIS are well documented *in vitro*, their physiological contribution *in vivo* remains uncertain, and distinguishing genuine tissue-level propagation from amplification artifacts will require improved senescence detection and lineage-resolved models [21]. Resolving these questions will be critical for understanding senescence heterogeneity and for translating senescence biology into precise therapeutic strategies.

Importantly, the heterogeneity of senescence inducers offers unique opportunities for selective targeting of senescent cells. Senolytics such as Dasatinib and Quercetin (D+Q) and BCL-2 family inhibitors show promise in clinical trials for age-related diseases, with inducer-specific engagement [99, 100]. For example, TIS cells from DNA damage inducers appear particularly sensitive to BCL-2 family inhibitors [100]. Specific senomorphics targeting SASP (e.g., IL-11 or IL-6 pathway inhibitors) may selectively suppress pro-tumorigenic secretory profiles from OIS or SIS without eliminating beneficial developmental senescence [99]. Pro-senescence therapies exploiting oncogene or replication stress pathways are advancing in clinical trials [101], though challenges remain in preventing SASP-mediated relapse and ensuring tissue-specific clearance. These inducer-tailored approaches promise greater precision but require improved biomarkers to distinguish senescence subtypes *in vivo* [102]. A deeper understanding of how distinct inducers shape senescent phenotypes will therefore be essential for rational design of precise senescence-targeting therapies.

More broadly, cellular senescence emerges from a broad spectrum of intrinsic and extrinsic stimuli, including DNA damage, telomere dysfunction, oncogenic signaling, oxidative and mitochondrial stress, cell–cell fusion, inflammatory cues, and programmed developmental signals. Although these inducers differ substantially, they converge on a limited set of core effector pathways, resulting in a stable proliferative arrest. Importantly, the nature of the initiating stimulus, together with cell type and the microenvironment settings, profoundly shapes the accompanying phenotypic features of senescence, including chromatin remodeling, metabolic rewiring, immune responses, and SASP composition and dynamics. This stimulus-dependent heterogeneity underlies the dualistic nature of senescence as both a beneficial and a detrimental biological program. Dissecting how distinct senescence-inducing cues are integrated into specific and context-dependent senescence phenotypes will be essential for developing therapeutic strategies that selectively eliminate deleterious senescent cells or modulate senescence and its downstream effects, thereby preserving the beneficial roles of senescence while limiting its long-term deleterious consequences in aging and disease.
